# *Talaromyces marneffei* Influences Macrophage Polarization and Sterilization Ability via the Arginine Metabolism Pathway in Vitro

**DOI:** 10.4269/ajtmh.21-0568

**Published:** 2022-07-25

**Authors:** Lin-xia Shen, Di Yang, Ri-feng Chen, Dong-hua Liu

**Affiliations:** ^1^ Department of Dermatology and Venereology, The First Affiliated Hospital of Guangxi Medical University, Nanning, People’s Republic of China;; ^2^ Department of Dermatology and Venereology, Huashan Hospital, Fudan University, Shanghai, People’s Republic of China;; ^3^ Department of Dermatology, The Third Affiliated Hospital of Guangxi Medical University, Nanning, People’s Republic of China

## Abstract

The opportunistic fungal pathogen *Talaromyces marneffei*, which is endemic across a narrow band of tropical Southeast Asia and southern China, is an intracellular pathogen that causes systemic and lethal infection through the mononuclear phagocyte system. The mechanisms by which *T. marneffei* successfully replicates and escapes the immune system remain unclear. To investigate the role of arginine metabolism in the escape of *T. marneffei* from killer macrophages, we assessed inducible nitric oxide synthase (iNOS) and arginase expression, nitric oxide (NO) production, arginase and phagocytic activity, and the killing of *T. marneffei* in a coculture system. Our results indicate that *T. marneffei* induced macrophage polarization toward the M2 phenotype and regulated the arginine metabolism pathway by prolonging infection, thereby reducing antimicrobial activity and promoting fungal survival. Moreover, inhibiting *T. marneffei*–induced macrophage arginase activity with N^ω^-hydroxy-nor-arginine restored NO synthesis and strengthened fungal killing. These findings indicate that *T. marneffei* affects macrophage polarization and inhibits macrophage antimicrobial function via the arginine metabolism pathway.

## INTRODUCTION

Talaromycosis is caused by *Talaromyces marneffei,* which is a temperature-dependent, dimorphic, opportunistic fungal pathogen. Talaromycosis was rarely diagnosed in the past. However, because of the increased proportion of the population infected with HIV and the wide use of glucocorticoids and immunosuppressors, talaromycosis has been endemic across a narrow band of tropical Southeast Asia and southern China.^1^^–^^4^ The mechanism of *T. marneffei* infection is unclear. Hamilton et al.^5^ suggested that *T. marneffei* infection originates in the lung after inhalation of airborne conidia, which enter alveoli, where conidia are engulfed by macrophages after combining with laminin. Through the phagocyte system, *T. marneffei* spreads and can be fatal to immunocompromised and healthy individuals. However, the mechanisms by which *T. marneffei* replicates and escapes immune killing successfully are unclear.

Macrophages participate in innate and adaptive immunity and play an essential role in internal environmental homeostasis. Under different pathophysiological conditions, macrophages polarize and thus acquire distinct functional phenotypes.^6^ Classically activated macrophages with the M1 phenotype, induced by interferon-γ or lipopolysaccharide (LPS), express the proinflammatory mediators tumor necrosis factor (TNF)-α, interleukin (IL)-1, IL-6, nitric oxide (NO), and reactive oxygen species, which are key effectors of macrophage microbicidal activity. In contrast, alternatively activated macrophages with the M2 phenotype, induced by IL-4 or IL-13, express anti-inflammatory mediators that promote the T helper 2 cell response and tissue repair.

The key to macrophage polarization is the arginine metabolism pathway.^7^ Inducible nitric oxide synthase (iNOS) hydrolyzes arginine into NO and L-ornithine. As a key component of the innate immune response in macrophages, NO has a strong antimicrobial effect against pathogens, especially intracellular pathogens such as *Mycobacterium tuberculosis*,^8^ *Leishmania major*,^9^ and *Salmonella enterica*.^10^ Arginase is a competitive inhibitor of iNOS, which hydrolyzes arginine into urea and L-ornithine, limiting the excessive production of NO. Many pathogens (e.g., *Mycobacterium bovis*, *Streptococcus pneumonia, Trypanosoma cruzi*, *Leishmania tropica*, and *Leishmania major*) increase arginase activity directly or affect host arginase activity, leading to the consumption of the substrate arginine, inhibiting competitively the synthesis of NO, inducing macrophages toward M2 phenotype polarization, and promoting persistent infection.^11^^–^^14^ The specific arginase inhibitor N^ω^-hydroxy-nor-arginine (nor-NOHA) was shown to restore NO synthesis in macrophages and enhance antimicrobial activity.^15^^,^^16^

In our previous experiment, the expression of CD86, CD163, and arginase 1 (Arg1) in skin lesions of patients, as well as in the lung, spleen, and liver tissues of mice infected with *T. marneffei*, suggested that M2 polarization of macrophages predominated over M1 polarization (data not yet published). Therefore, we speculate that *T. marneffei* affects macrophage polarization. In this study, we investigated the effect of the arginine metabolism pathway on *T. marneffei* escape from killer macrophages.

## MATERIALS AND METHODS

### Cell infection model.

The *T*. *marneffei* strain FRR2161 was cultured in the hypha phase for 2 weeks at 25°C on potato dextrose agar medium (6 g potato extract, 20 g dextrose, 20 g agar). Colonies were placed in 1× phosphate-buffered saline solution and the hyphae were filtered through sterile gauze to obtain a conidia suspension. The RAW264.7 murine macrophage cell line obtained from American Type Culture Collection (VA) was maintained in complete Dulbecco’s modified Eagle’s medium containing 10% fetal bovine serum (Gibco), 100 U/mL penicillin, and 100 mg/mL streptomycin at 37°C in a humidified atmosphere containing 5% carbon dioxide. A model of in vitro *T. marneffei* infection of macrophages was constructed for our research. M1 polarization of macrophages was induced by treatment with 500 ng/mL LPS (Invitrogen) for 24 hours. Macrophages were infected with *T. marneffei* at a multiplicity of infection (MOI) of 10 at 37°C for 24, 48, and 72 hours, and macrophages in medium without *T. marneffei* were used as the control group. LPS-activated macrophages were preincubated with 20 μM nor-NOHA (Merck) before being cocultured with *T. marneffei* for 24 hours (MOI = 10). Cells and supernatant were collected for subsequent experiments.

### Real-time quantitative polymerase chain reaction.

Macrophages were collected with TRIzol reagent (Invitrogen). Total RNA was extracted and transformed into complementary DNA using a RevertAid First Strand complementary DNA synthesis kit (Roche, Switzerland). Hieff^TM^ real-time quantitative polymerase chain reaction SYBR^TM^ Green Master Mix (Yeasen Biotechnology, Shanghai, China) was used for real-time quantitative polymerase chain reaction analysis with a LightCycler 480 System (Roche, Switzerland). The following program was run: 95°C for 5 minutes, 40 cycles of 95°C for 10 seconds, and 60°C for 30 seconds. The data were normalized to glyceraldehyde-3-phosphate dehydrogenase and calculated by the 2^–ΔΔCT^ method. The primer sequences were listed in Table 1.

**Table 1:** Primer sequences used for real-time quantitative polymerase chain reaction

Gene	Forward sequence (5′-3′)	Reverse sequence (5′-3′)
GAPDH	GGTCGGTGTGAACGGATTTG	TGTAGACCATGTAGTTGAGGTCA
iNOS	GAGGCCCAGGAGGAGAGAGATCCG	TCCATGCAGACAACCTTGGTGTTG
TNF-α	CCAAAGGGATGAGAAGTTCC	CTCCACTTGGTGGTTTGCTA
IL-1β	TGGCAACTGTTCCTGAACTCAA	AGCAGCCCTTCATCTTTTGG
Arg1	AGCTCTGGGAATCTGCATGG	ATGTACACGATGTCTTTGGCAGATA
CD301	ACTGAGTTCCTGCCTCTGGT	ATCTGGGACCAAGGAGAGTG
IL-10	CCCAGAAATCAAGGAGCATT	TCACTCTTCACCTGCTCCAC

Arg1 = arginase 1; GAPDH = glyceraldehyde-3-phosphate dehydrogenase; iNOS = inducible nitric oxide synthase; IL = interleukin; TNF-α = tumor necrosis factor α.

### Western blotting.

The concentration of protein samples was measured using a Pierce bicinchoninic acid protein assay kit (Thermo). Equal amounts of protein were separated by 10% sodium dodecyl sulfate–polyacrylamide gel electrophoresis and subsequently transferred onto a polyvinylidene fluoride membrane. After blockade of nonspecific antigens with 5% skim milk for 1 hour, the polyvinylidene fluoride membrane was incubated with primary antibodies against Arg1 (1:1,000; ab124917, Abcam, UK), iNOS (1:1,000; ab178945, Abcam, UK), IL-4R (1:500; sc-165974, Santa Cruz), or CD86 (1:1,000; ab53004, Abcam, UK) at 4°C overnight and then incubated with secondary antibodies for 1.5 hours at room temperature. The target proteins were visualized with an enhanced chemiluminescence system (Amersham Imager 600, GE) and analyzed with ImageJ software. β-Actin-horseradish peroxidase (1:10,000; 12620S, Cell Signaling Technology) was used as the loading control.

### Arginase activity assay.

Arginase activity was determined using an arginase activity assay kit (Sigma-Aldrich). Samples were analyzed according to the manufacturer’s instructions. Activity, measured in units per liter, was determined as follows:

$$\frac{\text{A}430_{\text{sample}}-\text{A}430_{\text{blank}}}{\text{A}430_{\text{standard}}-\text{A}430_{\text{water}}}\times \frac{1\;\text{mM}\times 50\times 10^{3}}{V\times T}.$$

### NO production assay.

The amount of NO produced was determined with a kit to measure nitrite levels (Nanjing Jiancheng Bioengineering Institute, China). A precipitation prepared from cells in coculture was centrifuged and discarded. The supernatant was collected. Different reagents were added according to the manufacturer’s instructions. The absorbance was then measured at 550 nm with a microplate reader (SpectraMax13, Molecular Devices).

### Immunofluorescence staining.

Macrophages were seeded onto coverslips in a 12-well plate and cultivated overnight in complete Dulbecco’s modified Eagle’s medium containing 10% fetal bovine serum, 100 U/mL penicillin, and 100 mg/mL streptomycin at 37°C in a humidified atmosphere containing 5% carbon dioxide. After coculture with *T. marneffei* conidia for 72 hours, macrophages were washed in phosphate-buffered saline. Cells were fixed in 4% paraformaldehyde for 15 minutes, permeabilized in 0.1% Triton X-100, and blocked in 1% bovine serum albumin for 15 minutes. All samples were incubated with primary antibody against CD206 (1:200; ab64693, Abcam, UK) at 4°C overnight and then incubated with secondary antibody for 1 hour at room temperature. Nuclei were counterstained with 4′6-diamidino-2-phenylindole (1:500, Sigma) for 10 minutes.

### Phagocytic activity assay.

The *T. marneffei* conidia suspension was prestained with Calcofluor white (Sigma) and treated with 10% potassium hydroxide for 10 minutes. Macrophages were added to coverslips and pretreated with 20 μM nor-NOHA for 2 hours. After coincubation with the prestained conidia suspension (MOI = 10) for 2 hours at 37°C, the samples were washed gently to remove conidia that were not phagocytosed by macrophages. The cells were then fixed in 0.5% paraformaldehyde. Fluorescence in the cells was observed with a fluorescence microscope (IX71; Olympus, Japan). Phagocytic activity was calculated by the phagocytic index using the following formula: Phagocytic index = No. of intracellular *T. marneffei* conidia/No. of macrophages containing *T. marneffei* conidia.

### *T. marneffei* killing assay.

*T. marneffei* killing was evaluated by colony-forming unit measurement. LPS-activated macrophages were pretreated with 20 μM nor-NOHA for 2 hours. The cells were then cocultured with *T. marneffei* conidia for 24 hours (MOI = 1). After 24 hours of incubation, the supernatant was collected. Sterile water was added to each well, and the plate was shaken. The contents in the wells were mixed with the collected supernatant and then centrifuged. The supernatant was discarded and the precipitate was resuspended in sterile water. This fungal suspension was then diluted serially with sterile water and plated eventually (quintuplicate samples) on solid yeast extract peptone dextrose agar plates.

### Statistical analysis.

Data were analyzed with GraphPad (version 7.01). Unpaired Student’s *t-*tests were performed to analyze the difference between two groups. One-way analysis of variance was performed for comparisons of multiple groups, followed by Tukey-Kramer’s multiple comparison test. *P* values < 0.05 were considered to be significant, and data from at least three repeated experiments were shown as the mean ± SD.

## RESULTS

### *T. marneffei* affected the arginine metabolism pathway in macrophages.

**Figure 1.**
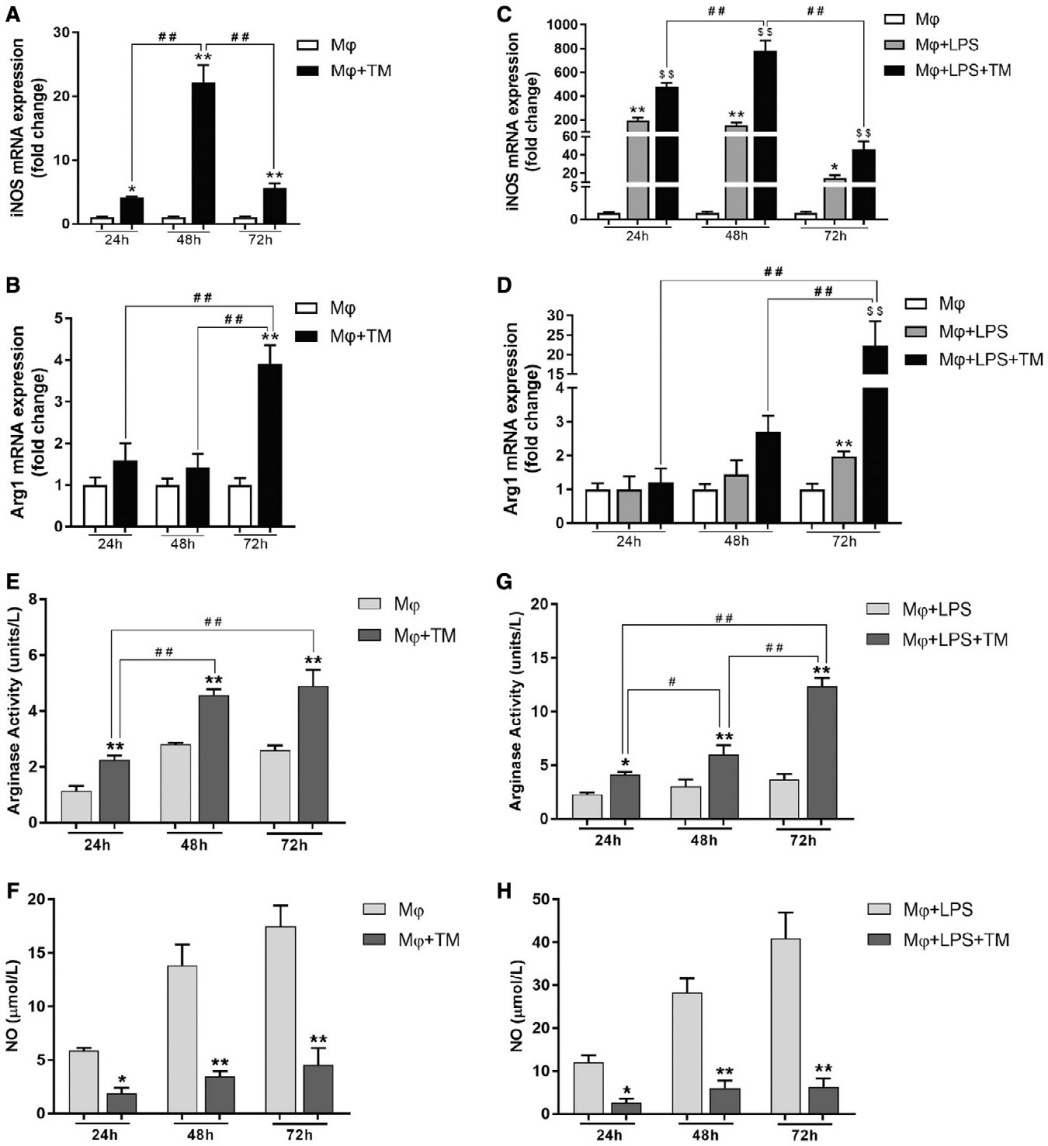
*Talaromyces marneffei* (TM) affected the transcriptional expression of inducible nitric oxide synthase (iNOS) and arginase 1 (Arg1) in macrophages. (**A, B**) Relative messenger RNA (mRNA) expression of iNOS and Arg1 in macrophages cocultured with TM conidia at 24, 48, and 72 hours (multiplicity of infection [MOI] = 10); macrophage (Mφ) + TM vs. Mφ, *P < 0.05, **P < 0.01; Mφ + TM vs. Mφ + TM, ^##^*P* < 0.01. (**C, D**) Relative mRNA expression of iNOS and Arg1 in lipopolysaccharide (LPS)-activated macrophages cocultured with TM conidia at 24, 48, and 72 hours (MOI = 10); Mφ + LPS vs. Mφ, *P < 0.05, **P < 0.01; Mφ + LPS + TM vs. Mφ + LPS, ^$$^*P* < 0.01; Mφ + LPS + TM vs. Mφ + LPS + TM, ^##^*P* < 0.01. *T. marneffei* affected the arginase activity and nitric oxide (NO) production in macrophages. (**E, F**) The arginase activity and NO production in macrophages cocultured with TM conidia at 24, 48, and 72 hours (MOI = 10); Mφ + TM vs. Mφ, *P < 0.05, **P < 0.01; Mφ + TM vs. Mφ + TM, ^##^*P* < 0.01. (**G, H**) The arginase activity and NO production in LPS-activated macrophages cocultured with TM conidia at 24, 48, and 72 hours (MOI = 10); Mφ + LPS + TM vs. Mφ + LPS, *P < 0.05, **P < 0.01; Mφ + LPS + TM vs. Mφ + LPS + TM, ^#^*P* < 0.05, ^##^*P* < 0.01.

**Figure 2.**
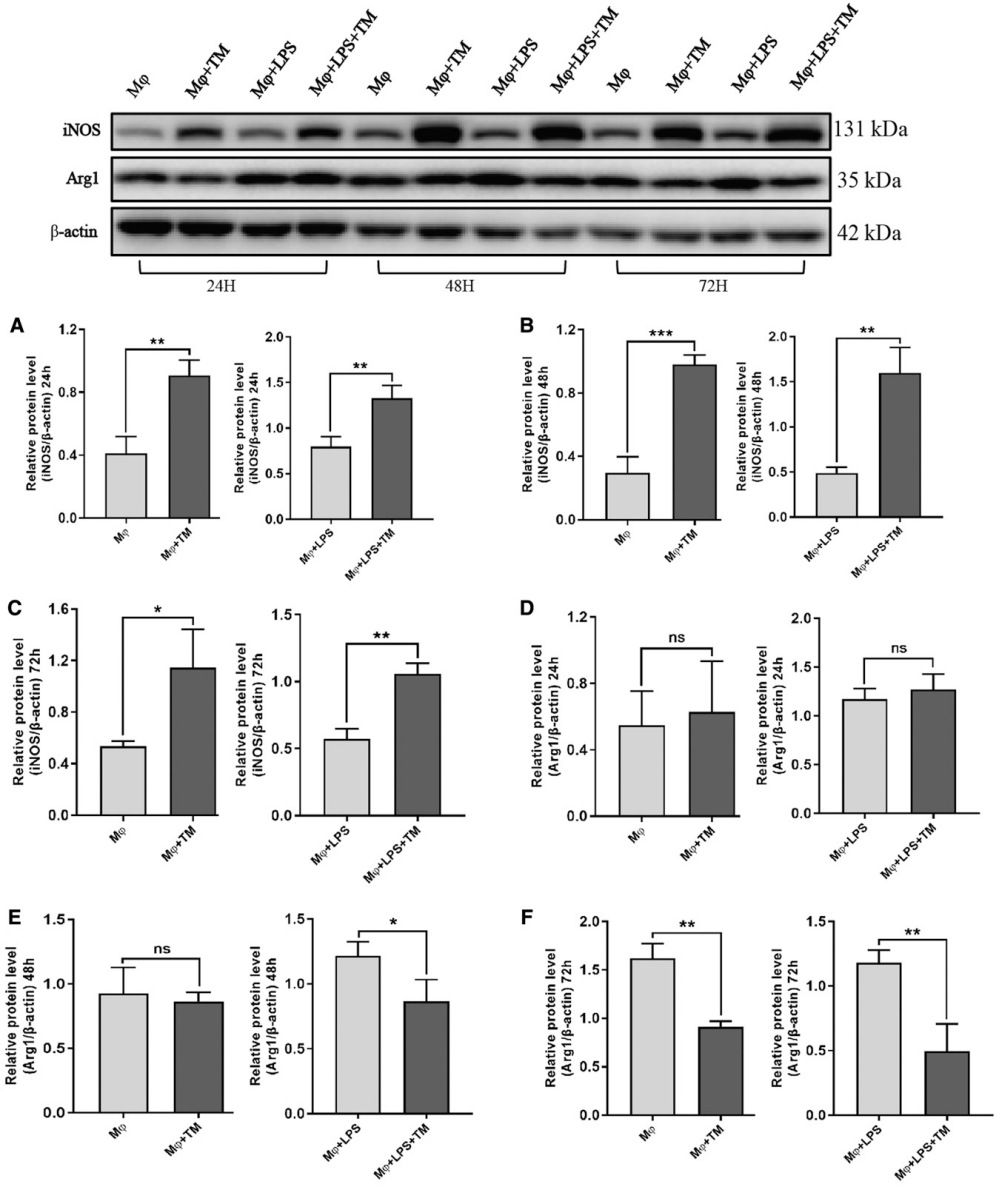
*Talaromyces marneffei* (TM) affected the protein expression of inducible nitric oxide synthase (iNOS) and arginase 1 (Arg1) in macrophages. (**A–C**) The iNOS protein expression in macrophages/lipopolysaccharide (LPS)-activated macrophages cocultured with TM conidia at 24, 48, and 72 hours, respectively (multiplicity of infection [MOI] = 10). (**D–F**) The Arg1 protein expression in macrophages/LPS-activated macrophages cocultured with TM conidia at 24, 48, and 72 hours, respectively (MOI = 10). *P < 0.05; **P < 0.01; ns = not significant. Mφ = macrophage.

We analyzed the messenger RNA (mRNA) and protein expression levels of iNOS and Arg1 in the coculture system. *T. marneffei* increased iNOS mRNA expression significantly. With prolonged coculture time, the iNOS mRNA level increased initially and then decreased after 72 hours (Figure 1A and C). However, Arg1 mRNA expression in the coculture system increased only after 72 hours (Figure 1B and D). *T. marneffei* increased iNOS protein expression significantly and similarly at 24, 48, and 72 hours (Figure 2A–C). However, *T. marneffei* decreased the Arg1 protein level in the Mφ + *T. marneffei *(TM) group at 72 hours and in the Mφ + LPS + TM group at 48 and 72 hours (Figure 2D–F). In addition, we examined the arginase activity of macrophages and the NO concentration in the supernatant. *T. marneffei* increased arginase activity substantially at 24, 48, and 72 hours. With prolonged coculture time, the arginase activity increased gradually (Figure 1E and G, Supplemental Table S1). NO synthesis in the coculture system decreased significantly at 24, 48, and 72 hours, but there was no significant difference among the 24-, 48-, and 72-hour time points (Figure 1F and H, Supplemental Table S2). In summary, the experiments indicated that *T. marneffei* affected the arginine metabolism pathway in macrophages.

### *T. marneffei* affected macrophage polarization.

**Figure 3.**
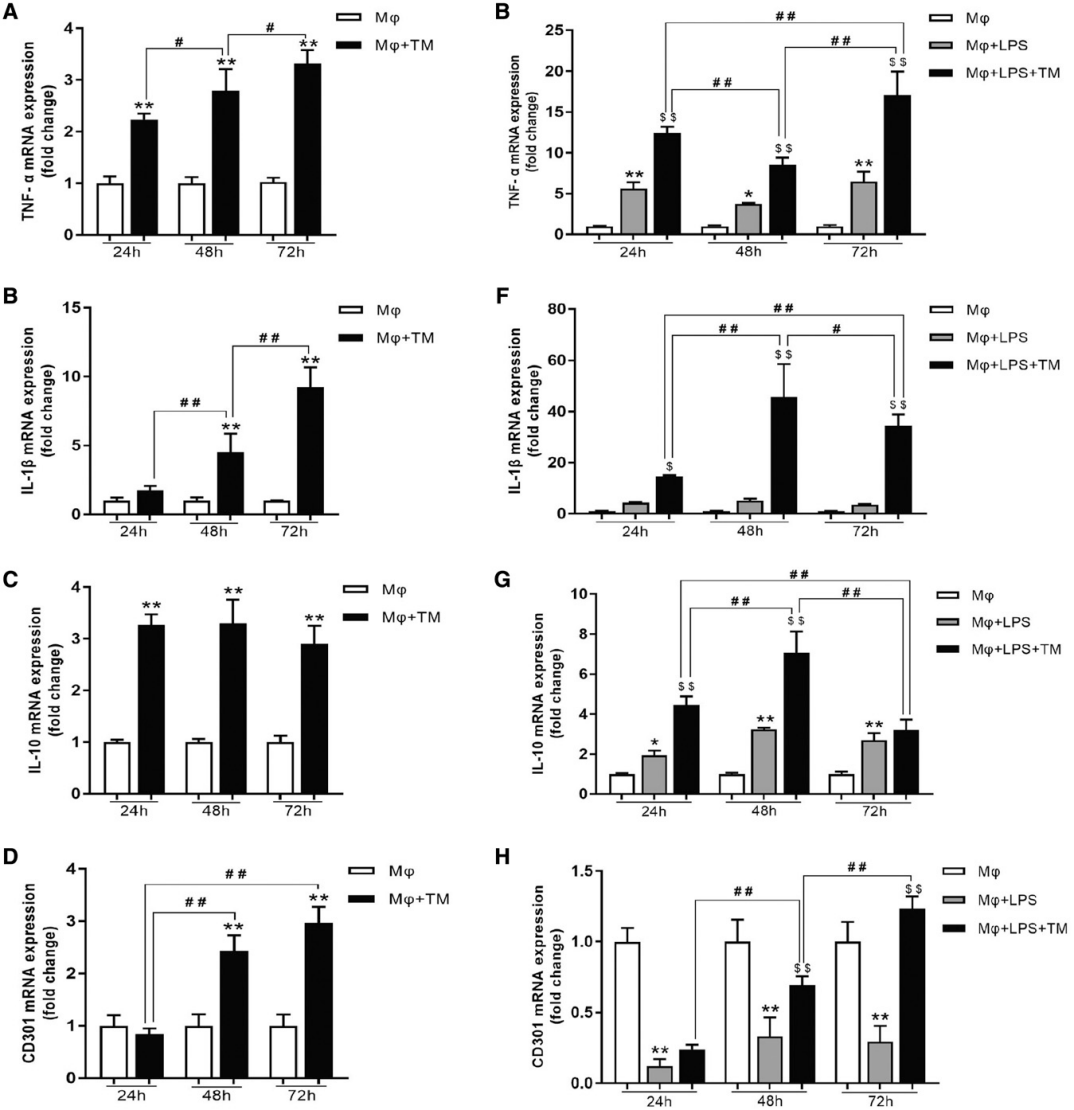
*Talaromyces marneffei* (TM) affected macrophage polarization. (**A**–**D**) Relative messenger RNA (mRNA) expression of tumor necrosis factor (TNF)-α, interleukin (IL)-1β, IL-10, and CD301 in macrophages cocultured with TM conidia at 24, 48, and 72 hours (multiplicity of infection [MOI] = 10); macrophage (Mφ) + TM vs. Mφ, **P < 0.01; Mφ + TM vs. Mφ + TM, ^#^*P* < 0.05, ^##^*P* < 0.01. (**E**–**H**) Relative mRNA expression of TNF-α, IL-1β, IL-10, and CD301 mRNA in lipopolysaccharide (LPS)-activated macrophages cocultured with TM conidia at 24, 48, and 72 hours (MOI = 10), Mφ + LPS vs. Mφ, *P < 0.05, **P < 0.01; Mφ + LPS + TM vs. Mφ + LPS, ^$^*P* < 0.05, ^$$^*P* < 0.01; Mφ + LPS + TM vs. Mφ + LPS + TM, ^#^*P* < 0.05, ^##^*P* < 0.01.

**Figure 4.**
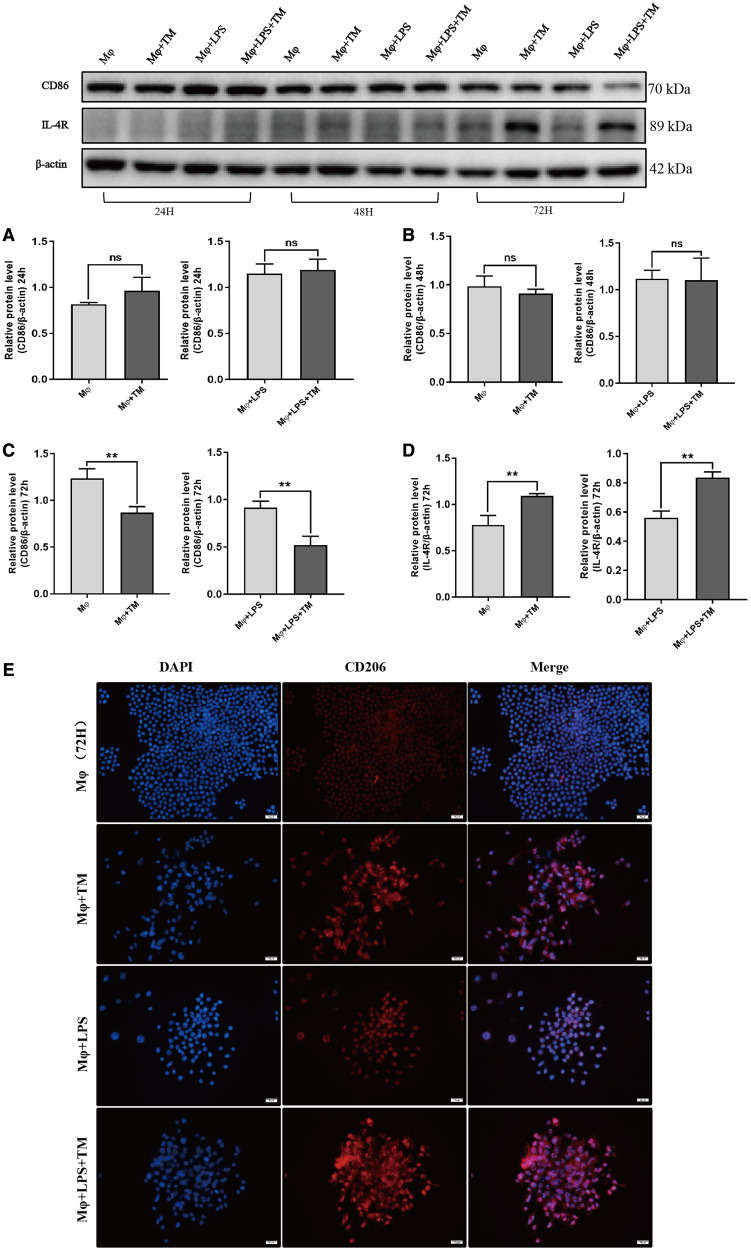
*Talaromyces marneffei* (TM) affected the protein expression of CD86, interleukin (IL)-4R, and CD206 in macrophages. (**A**–**C**) The CD86 protein expression in macrophages/lipopolysaccharide (LPS)-activated macrophages cocultured with TM conidia at 24, 48, and 72 hours respectively (multiplicity of infection [MOI] = 10). (**D**) The IL-4R protein expression in macrophages/LPS-activated macrophages cocultured with TM conidia at 72 hours (MOI = 10). **P < 0.01; ns = not significant. (**E**) The fluorescence analysis of CD206 in macrophages/LPS-activated macrophages cocultured with TM conidia at 72 hours (MOI = 10). DAPI = 4′6-diamidino-2-phenylindole. This figure appears in color at www.ajtmh.org.

To explore the effect of *T. marneffei* on macrophage polarization, we analyzed the mRNA expression of TNF-α, IL-1β, CD301, and IL-10 in the coculture system. *T. marneffei* increased significantly the mRNA expression of TNF-α and IL-1β in macrophages. With prolonged coculture time, the TNF-α and IL-1β mRNA expression in the Mφ + TM group increased gradually (Figure 3A and B). The TNF-α mRNA expression in the Mφ + LPS + TM group showed a fall–rise trend (Figure 3E), whereas the IL-1β mRNA expression increased initially and then decreased (Figure 3F). In addition, *T. marneffei* increased significantly the IL-10 mRNA level in the Mφ + TM group at 24, 48, and 72 hours, and there was no significant difference among the 24-, 48-, and 72-hour time points (Figure 3C). *T. marneffei* also elevated the IL-10 mRNA level in the Mφ + LPS + TM group at 24 and 48 hours (Figure 3G). CD301 mRNA expression in the coculture system increased gradually at 48 and 72 hours (Figure 3D and H). In addition, we analyzed CD86, IL-4R, and CD206 protein expression in the coculture system. CD86 is a marker of M1 polarization. IL-4R and CD206 are markers of M2 polarization. Our results showed that *T. marneffei* reduced CD86 protein expression at 72 hours, and increased IL-4R and CD206 protein expression at 72 hours (Figure 4). In summary, *T. marneffei* affected macrophage polarization and promoted macrophage polarization toward the M2 phenotype at 72 hours.

### Effect of nor-NOHA on the arginine metabolism pathway in the coculture system.

**Figure 5.**
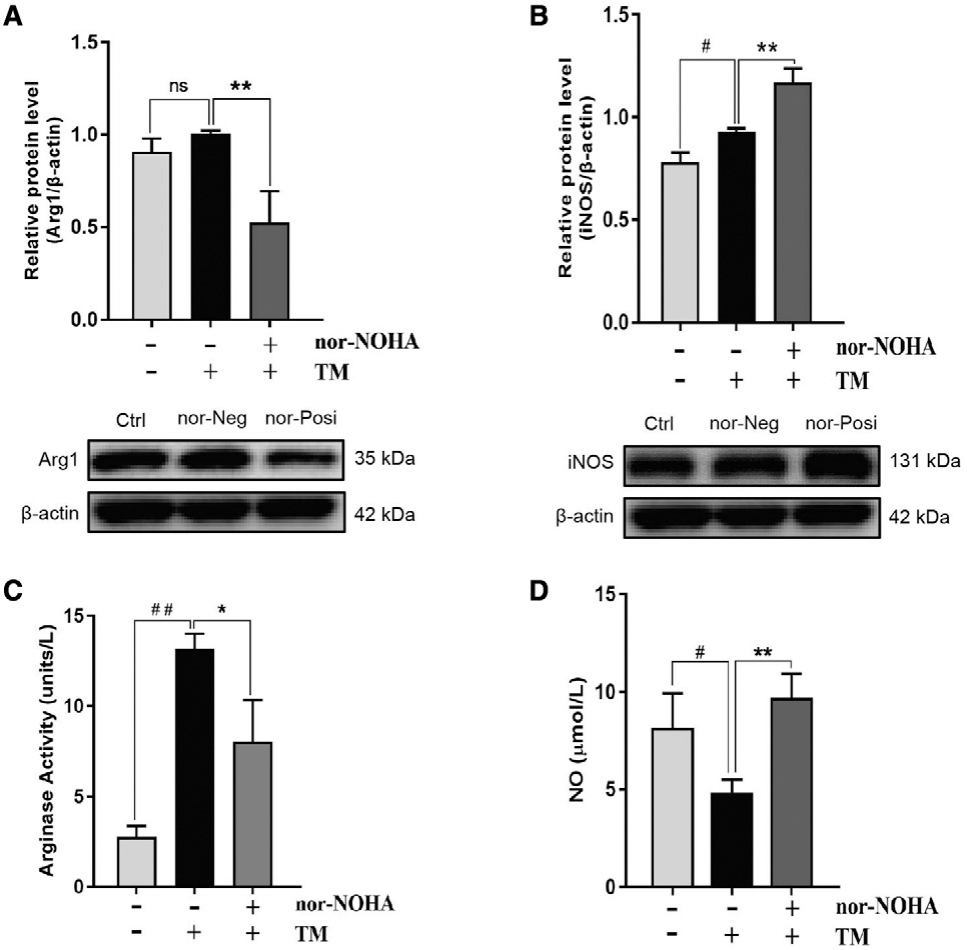
Effect of N^ω^-hydroxy-nor-arginine (nor-NOHA) on the arginine metabolism pathway in the coculture system. (**A**, **B**) The arginase 1 (Arg1) and inducible nitric oxide synthase (iNOS) protein expressions in lipopolysaccharide (LPS)-activated macrophages pretreated with and without 20 μM nor-NOHA before being cocultured with *Talaromyces marneffei* (TM) conidia at 24 hours (multiplicity of infection [MOI] = 10). (**C**, **D**) The arginase activity and nitric oxide (NO) production in LPS-activated macrophages pretreated with and without 20 μM nor-NOHA before being cocultured with TM conidia at 24 hours (MOI = 10); LPS-activated macrophages pretreated without 20 mM nor-NOHA (nor-Neg) vs. control (Ctrl), ^#^*P* < 0.05, ^##^*P* < 0.01; ns = not significant; LPS-activated macrophages pretreated with 20 mM nor-NOHA (nor-Posi) vs. nor-Neg, *P < 0.05, **P < 0.01.

The aforementioned experiments demonstrated that *T. marneffei* mediated macrophage polarization via the arginine metabolism pathway. Furthermore, we explored the effect of nor-NOHA, a specific arginase inhibitor, on macrophages in the coculture system. The results indicated that nor-NOHA decreased Arg1 protein expression significantly and increased iNOS protein expression (Figure 5A and B). In addition, the arginase activity of macrophages and NO level in the coculture supernatant were analyzed. Our results showed that 20 μM nor-NOHA inhibited substantially the *T. marneffei*–induced increase in arginase activity (Figure 5C) and reversed the *T. marneffei*–induced decrease in NO production (Figure 5D, Supplemental Table S3).

### nor-NOHA promoted the antimicrobial function of macrophages.

**Figure 6.**
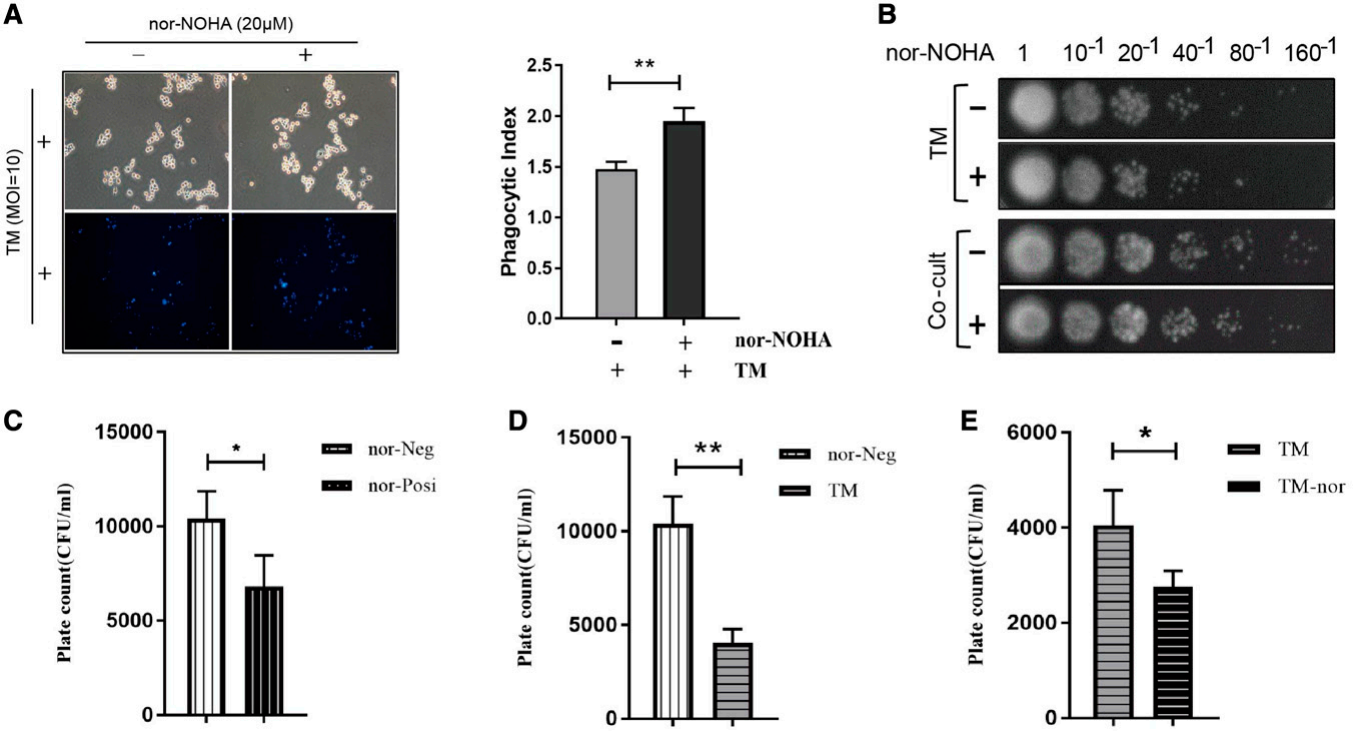
N^ω^-hydroxy-nor-arginine (nor-NOHA) promoted the antimicrobial function of macrophages. (**A**) Stained *Talaromyces marneffei* (TM) conidia with Calcofluor white and 10% potassium hydroxide. Lipopolysaccharide (LPS)-activated macrophages pretreated with and without 20 μM nor-NOHA for 2 hours before being cocultured with stained TM conidia (multiplicity of infection [MOI] = 10). The bright-blue TM conidia in the LPS-activated macrophages pretreated with 20 mM nor-NOHA (nor-Posi) group were more than those in the LPS-activated macrophages pretreated without 20 mM nor-NOHA (nor-Neg) group using the fluorescence microscope. The phagocytic index of macrophages was calculated; nor-Posi vs. nor-Neg, *P < 0.05. (**B**–**E**) TM conidia treated with and without 20 μM nor-NOHA for 2 hours; LPS-activated macrophages pretreated with and without 20 μM nor-NOHA for 2 hours before being cocultured with TM conidia at 24 hours (MOI = 1). Colony-forming units were calculated with the plate colony-counting method. Nor-Posi vs. nor-Neg, *P < 0.05; nor-Neg vs. TM, **P < 0.01; TM-nor vs. TM, *P < 0.05. This figure appears in color at www.ajtmh.org.

Because nor-NOHA increased the synthesis of NO in the coculture system, we determined the impact of nor-NOHA on the antimicrobial function of macrophages. Inhibition of arginase activity with 20 μM nor-NOHA increased significantly the intake of *T. marneffei* by macrophages and the killing of *T. marneffei* (Figure 6A–C, Supplemental Table S4). The colony count was obviously greater in the coculture system than in the *T. marneffei* group (Figure 6B and D). In addition, the colony count was less in the *T. marneffei*–only group treated with 20 μM nor-NOHA (Figure 6B and E). In summary, nor-NOHA enhanced the antimicrobial function of macrophages.

## DISCUSSION

*T. marneffei* is an intracellular fungus that mainly infects immunodeficient populations, such as HIV-positive and anti–interferon-γ autoantibody-positive patients.^17^^,^^18^ Macrophages are versatile cells involved predominantly in host defense and immunity. Classically activated macrophages increase the production of proinflammatory cytokines, reactive oxygen species, and nitrogen species, enabling efficient killing of phagocytosed microbes. Our study showed that the expression of the proinflammatory factors TNF-α and IL-1β was increased significantly in macrophages after a *T. marneffei* treatment. In addition, the levels of the anti-inflammatory factors IL-10 and CD301 were elevated. In contrast, alternatively activated macrophages promoted the synthesis of anti-inflammatory and tissue repair factors. This finding indicates that the *T. marneffei*–induced immune response is a complex and dynamic process in macrophages.

Macrophages have a broad array of cell surface receptors and intracellular mediators that regulate the recognition, phagocytosis, and destruction of pathogens. CD86 is a member of the Ig superfamily that is expressed mainly on antigen-presenting cells such as dendritic cells and mononuclear macrophages. CD86, along with CD80, provide costimulatory signals necessary for T-lymphocyte proliferation and activation, which play vital roles in clearing pathogens and maintaining the balance of the immune system.^19^ CD86 is also a surface marker of macrophage M1 polarization. In our previous study,^20^ we found that both macrophages and *T. marneffei* yeast cells expressed CD86 in the skin tissue of patients. Moreover, previous in vitro experiments showed that the protein level of CD86 in macrophages decreased after human THP-1 cells were cocultured with *T. marneffei* for 72 hours.^21^ This finding was consistent with our current result that CD86 expression in murine macrophages was reduced by a *T. marneffei* treatment of 72 hours. Therefore, we believe the *T. marneffei*–induced M1 polarization of macrophages decreases gradually with the extension of the coculture period.

To determine whether *T. marneffei* promoted M2 polarization of macrophages, we measured the expression of CD206 and IL-4R. CD206, also known as the mannose receptor, mediates the internalization of viruses, bacteria, and fungi; facilitates antigen uptake and processing in the adaptive immune response; and mediates the direct uptake of pathogens in the innate immune response.^22^ Activation of CD206 can affect the maturation of phagosomes and provide favorable conditions for the survival of pathogens within macrophages by restricting the fusion of phagosomes and lysosomes.^23^^,^^24^ CD206 is also a surface marker of macrophage M2 polarization. Our results showed that the protein expression of CD206 was increased significantly by a *T. marneffei* treatment of 72 hours, indicating that macrophages favored M2 polarization, facilitating the survival of pathogens. This alternatively activated phenotype can be induced by the canonical type 2 cytokine IL-4. IL-4R is a specific receptor of IL-4 and a surface marker of M2 polarization. After binding to IL-4R, IL-4 exerts an immunosuppressive effect via the STAT6 signaling pathway.^25^ Our results showed that the expression of IL-4R was increased significantly by a *T. marneffei* treatment of 72 hours, further demonstrating that *T. marneffei* induces macrophage switching from the M1 to the M2 phenotype, which results in blunted pathogen killing.

The arginine metabolism pathway is essential to macrophage polarization. Our results show that *T. marneffei* increased the mRNA and protein expression of iNOS in macrophages, but decreased NO production in the coculture system. In addition, we found an opposite trend between the mRNA and protein expression of Arg1 in the macrophages cocultivated with *T. marneffei* for 72 hours, and arginase activity was increased. Given these interesting results, we speculate that *T. marneffei* enhances the arginase activity of macrophages, causing an extensive arginase reaction and consumption of the substrate L-arginine and leading to decreased Arg1 protein levels within 72 hours. The presence of arginase, a competitive binder of L-arginine, inhibits iNOS binding with L-arginine, resulting in significantly reduced NO synthesis in macrophages. Therefore, *T. marneffei* affects the arginine metabolism pathway of macrophages and inhibits NO production, thus attenuating the antibiotic function of macrophages.

Recent studies have shown upregulated expression of arginase in macrophages infected with fungi, parasites, and bacteria.^26^^–^^28^ As a specific inhibitor of arginase, nor-NOHA can inhibit arginase activity effectively without inhibiting the upregulation of iNOS expression. The application of nor-NOHA has been studied in patients with hypertension, coronary disease, heart failure, and familial hypercholesterolemia, and has led to outstanding results.^29^^–^^32^ However, few studies have investigated nor-NOHA as a treatment of fungal infection. Our experiments indicate that nor-NOHA inhibits arginase activity and increases the synthesis of NO. In addition, nor-NOHA enhanced *T. marneffei* phagocytosis by macrophages, restoring the killing action of macrophages. This outcome demonstrates that nor-NOHA might be an effective agent for treating *T. marneffei* infection.

In addition, we found that the number of colonies in the cocultured system was significantly greater than that in the *T. marneffei* group. The reason why macrophages promote the proliferation of *T. marneffei* is unknown. Moreover, our results showed that nor-NOHA repressed the proliferation of *T. marneffei* without macrophage involvement. Whether the arginine metabolism pathway exists in *T. marneffei* and the reason why nor-NOHA affects the reproduction of *T. marneffei* without macrophage involvement remain unclear. Moreover, only one reference strain, *T. marneffei* FRR2161, was used in the study. Further studies, including in vivo murine experiments and analyses of clinical isolates, are needed. In our current work, we explored the mechanism by which *T. marneffei* escapes macrophage killing and suggest that nor-NOHA might be a promising therapeutic agent for *T. marneffei* infection.

## CONCLUSION

*T. marneffei* FRR2161 enhanced the arginase activity of macrophages and decreased NO synthesis in vitro. It alleviated antimicrobial function by promoting macrophage polarization toward the M2 phenotype. The use of nor-NOHA, a specific inhibitor of arginase, might be an effective strategy for treating *T. marneffei* FRR2161 infection.

## Supplemental files


Supplemental materials
