# Supplementary material for: Talaromyces marneffei Influences Macrophage Polarization and Sterilization Ability via the Arginine Metabolism Pathway in Vitro
Source: Am J Trop Med Hyg. 2022 Jul 25;107(3):600–9. doi: 10.4269/ajtmh.21-0568 (PMC9490654; doi:10.4269/ajtmh.21-0568)
Supplement: Supplementary file 1 [file tpmd210568.SD1.pdf]

Supplemental Table S1. Fig1 E and G

Group	24h (units/L)	48h (units/L)	72h (units/L)
Mφ	1.13±0.19	2.80±0.06	2.60±0.16
Mφ+TM	2.25±0.15	4.58±0.19	4.90±0.57
Mφ+LPS	2.29±0.17	3.04±0.63	3.66±0.54
Mφ+LPS+TM	4.14±0.26	5.99±0.88	12.39±7.03

Supplemental Table S2. Fig1 F and H

Group	24h (μmol/L)	48h (μmol/L)	72h (μmol/L)
Mφ	5.86±0.26	13.83±1.95	17.49±1.95
Mφ+TM	1.89±0.52	3.46±0.50	4.54±1.56
Mφ+LPS	12.01±1.67	28.32±3.29	40.89±6.05
Mφ+LPS+TM	2.65±0.88	5.90±1.86	6.30±1.96

Supplemental Table S3. Fig 5 C and D

Group	Arginase activity (units/L)	NO production (μmol/L)
Ctrl	2.78±0.60	8.17±1.76
nor-Neg	13.18±0.84	4.83±0.67
nor-Posi	8.06±2.29	9.70±1.24

Supplemental Table S4. Fig 6

Group	Phagocytic Index	Colony-Forming Unit (CFU/ml)
nor-Neg	1.48±0.07	10400±1461.00
nor-Posi	1.95±0.13	6800±1665.00
TM	/	4050±737.10
TM-nor	/	2750±341.60
